# Renal mitochondrial toxicity: effects of thymidine analogues and tenofovir disoproxil fumarate in African people living with HIV

**DOI:** 10.1097/QAD.0000000000003209

**Published:** 2022-03-04

**Authors:** Matthew Hunt, Richard Phillips, Yasmine Hardy, Dorcas O Owusu, Rosa Mitchelmore, Mehrab Durrani, Brendan AI Payne, David R Chadwick

**Affiliations:** 1 Wellcome Centre for Mitochondrial Research, Translational and Clinical Research Institute, Newcastle University, Newcastle-upon-Tyne, UK; 2 Dermatology and Venereology Division, Department of Medicine (Solna), Karolinska Institutet, Stockholm, Sweden; 3 Department of Medicine, Kwame Nkrumah University of Science and Technology, Kumasi, Ghana; 4 Department of Medicine, Komfo Anokye Teaching Hospital, Kumasi, Ghana; 5 Kumasi Centre for Collaborative Research in Tropical Medicine, Kumasi, Ghana; 6 School of Medicine, Newcastle University, Newcastle-upon-Tyne, UK; 7 Department of Infection and Tropical Medicine, Royal Victoria Infirmary, Newcastle-upon-Tyne, UK; 8 Centre for Clinical Infection, James Cook University Hospital, Middlesbrough, UK

**Keywords:** Tenofovir, renal insufficiency, mitochondrial DNA, anti-retroviral therapy, HIV

## Abstract

We investigated the contributions of thymidine analogue (TA) and tenofovir disoproxil fumarate (TDF) anti-retroviral therapy on renal mitochondrial toxicity in Ghanaian PWH. Similar levels of renal biochemical and mitochondrial dysfunction were seen, and there was no increased risk in PWH who had sequenced from TA to TDF. However, mild renal impairment was associated with mitochondrial DNA damage in TDF but not TA-treated PWH. These data support the continued use of TDF in resource limited settings.

Tenofovir disoproxil fumarate (TDF) is commonly used in anti-retroviral therapy (ART). It remains part of first-line ART in WHO guidelines for low and middle-income countries (LMIC) [1]. Whilst TDF is generally well tolerated, renal complications are well recognised in people with HIV (PWH). These include deterioration in estimated glomerular filtration rate (eGFR) and proximal tubular dysfunction, including renal Fanconi syndrome [2, 3]. Older nucleoside analogue reverse transcriptase inhibitors (NRTIs) including the thymidine analogues (TA), zidovudine (AZT) and stavudine (d4T), have previously been shown to inhibit the mitochondrial DNA (mtDNA) polymerase, pol-*γ* [4, 5]. This results in systemic mitochondrial toxicities affecting various tissues such as myopathy [6] and peripheral neuropathy [4, 7]. AZT remains commonly used in many LMIC [8]. TDF has a low affinity for pol-*γ* and is not considered to show systemic mitochondrial toxicity [9, 10]. Nevertheless, it is suspected that TDF-induced renal abnormalities are the result of a toxic accumulation of the active metabolite in proximal tubular cells, sufficient to induce mitochondrial defects [11]. A recent study by our group demonstrated the presence of the mtDNA ‘common deletion’ mutation (CD) in urine samples from TDF-treated PWH in the UK [12]. The mtDNA CD is the commonest somatic mtDNA mutation seen in ageing and has also been detected in the setting of exposure to older NRTIs with systemic mitochondrial toxicity [13–16]. Given that sequential exposure to a TA followed by TDF is very common in LMIC, we hypothesised that this might result in a ‘double-hit’ of renal mtDNA damage.

PWH attending the HIV clinic at Komfo Anokye Teaching Hospital in Kumasi, Ghana and who had been taking ART for at least 3 years were recruited in 2020. All participants gave written informed consent and the study was approved by local research ethics committee. Participants with known (non-TDF associated) renal disease were excluded. Paired serum and urine samples were collected for renal biochemistry. Patients with haematuria were subsequently excluded. An additional urine sample was collected for mtDNA analysis. Real-time PCR was performed on the urine cellular pellet as previously described [12].

The study population comprised 97 participants of which 74% were female, and mean age was 49 years. 39% were on an ART regimen that included TDF (TDF^+^, n 38), with a median of 81 months TDF exposure. Of the TDF+ participants, 68% had switched from ART that included a TA (TDF+/TA+, n 26). The non-TDF treated group were all on a regimen that included AZT (TDF^-^/TA^+^, n 59). Full study population clinical characteristics are described in Supplementary Table 1. eGFR, fractional excretion of phosphate (FEPi) and proteinuria did not differ by ART regimen.

There was no significant difference in mtDNA content (copy number, CN) by ART exposure (Supplementary Table 1, Figure 1A). MtDNA CN was not significantly correlated with age (r 0.07, p 0.48), duration on ART (r 0.04, p 0.72), eGFR (r -0.11, p 0.30), or FEPi (r 0.21, p 0.11). The mtDNA CD mutation was detected in 33/96 urine samples (34%). 19 (32%) TDF^-^/TA^+^, 4 (36%) TDF^+^/TA^-^ and 10 (40%) TDF^+^/TA^+^ participants had detectable urine mtDNA CD (Figure 1B). Amongst TDF-treated subjects, the presence of the mtDNA CD predicted lower eGFR (Supplementary Table 2, Figure 1C). 64% of TDF-treated subjects with stage 2 or 3 CKD had detectable mtDNA CD in urine compared with only 25% of TDF-treated subjects with normal renal function (p 0.022). This effect was not seen in TDF^-^/TA^+^ subjects. TDF^+^/CD^+^ and TDF^+^/CD^-^ participants did not differ by age, duration of TDF exposure, FEPi or proteinuria.

Few studies have examined the role of mitochondrial toxicity of ART in LMIC settings, and none in the context of ART-induced renal dysfunction. TA therapy was the basis of initial ART rollout in LMIC, being progressively replaced by TDF in more recent years. Here we have compared TA therapy, known to have systemic mitochondrial toxicity, with TDF therapy, thought to have toxicity limited to the renal tract. In addition, we specifically addressed whether sequential exposure to a TA followed by TDF might increase the risk of TDF-associated renal toxicity through a mitochondrial ‘double-hit’ mechanism. In keeping with our previous UK study [12], we detected the mtDNA common deletion mutation in about one third of subjects, but this did not differ by ART regimen. As expected, and reflecting the evolution of ART in LMIC, those subjects exposed to TDF had a shorter duration of diagnosed HIV infection and of total ART exposure than those receiving TA-based ART. It is very plausible that TDF-induced renal mtDNA damage may be cumulative over time, and it will be important to perform longitudinal studies of this nature to confirm the longer term safety of TDF. We did not observe any excess of renal biochemical or mitochondrial dysfunction in those TDF-treated subjects who had prior TA exposure, arguing against the hypothesised ‘two-hit’ model for TDF-induced renal mitochondrial damage. Nevertheless, the presence of the mtDNA CD was significantly associated with lower eGFR in TDF-treated subjects. This renal impairment was generally mild (CKD stage 2), but suggests that renal-specific mtDNA damage may contribute to TDF-associated renal dysfunction. In contrast, this pattern was not seen in TDF-unexposed TA-treated subjects, suggesting a specificity of TDF for renal mtDNA damage.

In summary, we detected evidence of mtDNA damage in the renal tract of ART-treated Ghanaian PWH, with TA and with TDF exposure. Sequential TA and TDF treatment did not increase the prevalence of mtDNA damage. Whilst TDF therapy appears generally well tolerated in this setting, we nevertheless observed that the presence of mtDNA mutation in the renal tract was associated with mild renal impairment in TDF-treated PWH. Overall, these data support the continued use of TDF for most PWH in LMIC, but reinforce the need for more longitudinal data. As TDF is associated with both renal and bone toxicity, there is a likely need for TAF (tenofovir alafenamide) for selected PWH in LMIC.

## Supplementary Material

Supplementary tables

## Figures and Tables

**Figure 1:**
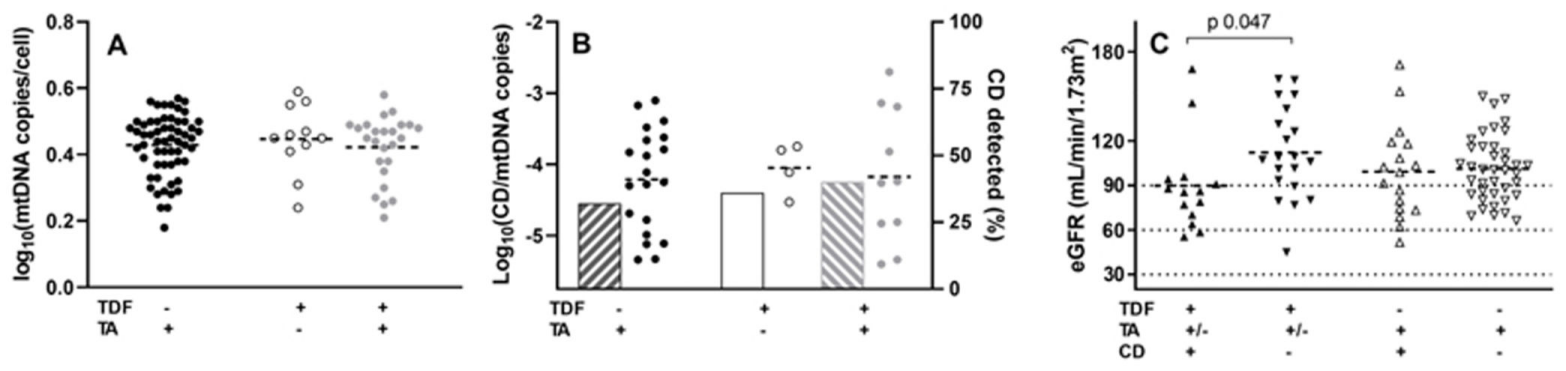
Urinary mtDNA. **(A)** mtDNA content in the urine of cART treated subjects. Line at mean. **(B)** mtDNA CD (common deletion) mutation detected in the urine of cART treated subjects. Right y-axis and columns shows proportion of subjects with CD detected. Left y-axis and dots shows levels of CD where detected. Line at mean. **(C)** eGFR in cART treated subjects with and without detectable mtDNA CD mutation in urine. X-axis for all panels shows exposure to tenofovir disoproxil fumarate (TDF) and thymidine analogues (TA), and in panel (C), the presence or absence of the mtDNA CD.
