# Supplementary material for: Renal mitochondrial toxicity: effects of thymidine analogues and tenofovir disoproxil fumarate in African people living with HIV
Source: AIDS. Author manuscript; Available in PMC 2022 Jun 13. (PMC7612849; doi:10.1097/QAD.0000000000003209)
Supplement: Supplementary tables [file EMS145823-supplement-Supplementary_tables.pdf]

Supplementary tables

	TDF$^-$/TA$^+$ n 59	TDF$^+$/TA$^-$ n 12	TDF$^+$/TA$^+$ n 26
**Age, mean years (SD)**	50 (10)	46 (10)	49 (7)
**Female gender, n (%)**	44 (75%)	9 (75%)	21 (73%)
**Black African ethnicity, n (%)**	59 (100%)	12 (100%)	26 (100%)
**BMI, median kg/m^2 (IQR)**	26.6 (22.9-32.2)	24.7 (23.0-30.9)	28.7 (22.9-36.7)
**Current smoker, n (%)**	3 (5%)	1 (8%)	0 (0%)
**Duration HIV diagnosis, median months (IQR)**	155 (131-173)	80 (31-103)	141 (116-150)
**cART duration, median months (IQR)**	145 (123-163)	56 (30-97)	139 (114-146)
**TDF duration, median months (IQR)**	-	64 (30-96)	86 (3-99)
**AZT duration, median months (IQR)**	132 (108-168)	-	48 (0-108)
**d4T duration, median months (IQR)**	0 (0-6)	-	0 (0-24)
**Boosting agents, n (%)**	4 (7%)	0 (0%)	2 (8%)
**CD4 count, median cells/µL (IQR)**	243 (127-311)	212 (85-333)	108 (38-251)
**Hepatitis B coinfection, n (%)**	2 (3%)	1 (8%)	1 (4%)
**Type 2 diabetes mellitus, n (%)**	3 (5%)	1 (8%)	2 (8%)
**Hypertension, n (%)**	11 (19%)	2 (18%)	7 (27%)
**eGFR, mean (SD) mL/min/1.73m^2**	100.7 (25.3)	107.8 (34.0)	101.7 (32.2)
**CKD Stage 1, n (%)**	36 (64%)	9 (75%)	13 (54%)
**CKD Stage 2, n (%)**	20 (35%)	2 (17%)	10 (42%)
**CKD Stage 3, n (%)**	1 (1%)	1 (8%)	1 (4%)
**Serum phosphate, mean mmol/L (SD)**	1.18 (0.28)	1.20 (0.33)	1.09 (0.24)
**FEPi, median % (IQR)**	5.46 (3.48-9.63)	6.66 (4.19-8.52)	5.52 (3.80-12.68)
**Hypophosphataemia, n (%)**	5 (11%)	0 (0%)	2 (10%)
**Proteinuria, n (%)**	5 (9%)	3 (25%)	1 (4%)
**Log_{10}(mtDNA copies/cell), median (IQR)**	0.45 (0.37-0.50)	0.45 (0.41-0.55)	0.45 (0.37-0.49)
**mtDNA common deletion (CD) detected, n (%)**	19 (32%)	4 (36%)	10 (40%)
**Log_{10}(CD/mtDNA copies), mean (SD)**	-4.21 (0.72)	-4.05 (0.36)	-4.17 (0.95)

Supplementary Table 1 – Cohort characteristics. TDF, tenofovir disoproxil fumarate; TA, thymidine analogue (d4T [stavudine], AZT [zidovudine]); BMI, body mass index; cART, combination anti-retroviral therapy; eGFR, estimated glomerular filtration rate (CKD-EPI); CKD, chronic kidney disease; FEPi, fractional excretion of phosphate index.

Author accepted manuscript

	TDF^+/CD^+	TDF^+/CD^-	p-value
**Age, mean years (SD)**	49 (9)	48 (8)	0.67
**BMI, mean kg/m² (SD)**	31.8 (12.7)	26.9 (7.1)	0.16
**Duration HIV diagnosis, mean months (SD)**	119 (46)	123 (48)	0.79
**cART duration, mean months (SD)**	108 (44)	115 (45)	0.63
**TDF duration, median months (IQR)**	76 (5-100)	81 (28-96)	0.75
**AZT duration, median months (IQR)**	6 (0-99)	0 (0-81)	0.86
**d4T duration, median months (IQR)**	0 (0-12)	0 (0-15)	0.73
**Boosting agents, n (%)**	0 (0%)	2 (9%)	0.51
**CD4 count, median cells/µL (IQR)**	148 (51-319)	168 (43-263)	0.96
**eGFR, mean (SD) mL/min/1.73m²**	89.9 (31.5)	112.2 (31.1)	**0.049**
**CKD Stage 1, n (%)**	5 (36%)	15 (75%)	**0.035**
**CKD Stage 2, n (%)**	8 (57%)	4 (20%)	**0.014**
**CKD Stage 3, n (%)**	1 (7%)	1 (5%)	0.17
**Serum phosphate, mean mmol/L (IQR)**	1.07 (0.29)	1.12 (0.26)	0.64
**FEPi, median % (IQR)**	6.9 (4.4-14.1)	5.9 (3.2-9.6)	0.33
**Hypophosphataemia, n (%)**	1 (11%)	1 (5%)	0.53
**Proteinuria, n (%)**	1 (7%)	2 (9%)	0.99
**Log_{10}(mtDNA copies/cell), median (IQR)**	0.44 (0.38-0.48)	0.46 (0.34-0.50)	0.60

Supplementary Table 2 – Characteristics of subjects with the mtDNA common deletion. TDF, tenofovir disoproxil fumarate; CD, mtDNA common deletion mutation; BMI, body mass index; cART, combination anti-retroviral therapy; AZT, zidovudine; d4T, stavudine; eGFR, estimated glomerular filtration rate (CKD-EPI); FEPi = fractional excretion of phosphate index.

Author accepted manuscript
